# Index of CD34+ Cells and Mononuclear Cells in the Bone Marrow of Spinal Cord Injury Patients of Different Age Groups: A Comparative Analysis

**DOI:** 10.1155/2012/787414

**Published:** 2012-07-05

**Authors:** Vidyasagar Devaprasad Dedeepiya, Yegneswara Yellury Rao, Gosalakkal A. Jayakrishnan, Jutty K. B. C. Parthiban, Subramani Baskar, Sadananda Rao Manjunath, Rajappa Senthilkumar, Samuel J. K. Abraham

**Affiliations:** ^1^Division of Translational Medicine, Nichi-In Centre for Regenerative Medicine (NCRM), C 16 & 17, Vijaya Health Centre Premises, 175 NSK Salai, Vadapalani, Chennai-600026, Tamil Nadu, India; ^2^Department of Biotechnology, Acharya Nagarjuna University, Nagarjuna Nagar, Guntur 522 510, India; ^3^Department of Medicine, KG Hospital, Arts College Road, Coimbatore 641018, India; ^4^Department of Cardiothoracic Surgery, Omega Hospital, Mangalore, Mahaveera Circle, Kankanady, Mangalore 575002, India; ^5^Department of Surgery, Faculty of Medicine, University of Yamanashi, 1110 Shimokato, Yamanashi, Chuo 409-3898, Japan

## Abstract

*Introduction*. Recent evidence of safety and efficacy of Bone Marrow Mononuclear Cells (BMMNC) in spinal cord injury makes the Bone Marrow (BM) CD34+ percentage and the BMMNC count gain significance. The indices of BM that change with body mass index and aging in general population have been reported but seldom in Spinal Cord Injury (SCI) victims, whose parameters of relevance differ from general population. Herein, we report the indices of BMMNC in SCI victims. *Materials and Methods*. BMMNCs of 332 SCI patients were isolated under GMP protocols. Cell count by Trypan blue method and CD34+ cells by flow cytometry were documented and analysed across ages and gender. *Results*. The average BMMNC per ml in the age groups 0–20, 21–40, 41–60, and 61–80 years were 4.71, 4.03, 3.67, and 3.02 million and the CD34+ were 1.05%, 1.04%, 0.94%, and 0.93% respectively. The decline in CD34+ was sharp between 20–40 and 40–60 age groups. Females of reproductive age group had lesser CD34+. *Conclusion*. The BMMNC and CD34+ percentages decline with aging in SCI victims. Their lower values in females during reproductive age should be analysed for relevance to hormonal influence. This study offers reference values of BMMNC and CD34+ of SCI victims for successful clinical application.

## 1. Introduction

With a reported global prevalence ranging from 236 to 1009 per million [1], Spinal Cord Injury (SCI) continues to be a devastating problem with no definite solutions. Spinal Cord Injury may be due to both traumatic (e.g., road traffic accidents) or nontraumatic causes (e.g., infections, congenital causes, tumours, etc.). In traumatic spinal cord injury, primary injury caused by compression or traction causes direct injury to neural elements due to the displaced bone fragments, ligaments, and disc material which leads to damage of the axons, neural cell bodies, and blood vessels. The spinal cord swells occupying the entire diameter of the spinal canal and ischemia results. The ischemia by releasing toxins gives rise to a cascade of secondary events ultimately leading to damage of the neighbouring healthy neurons [2]. The current mainline approaches of treatment involve removal of the bone fragments or other components to decompress the swollen spinal cord with the primary approach being limiting the secondary damage, followed by rehabilitation to assist in spontaneous recovery [2]. However the recovery is only limited in most of the cases. Hence, newer therapeutic options are being explored which might aid in complete recovery of the injured spinal cord. In this context, in addition to pharmacological treatment for improving the regeneration of the neurons using antiapoptotic agents, growth factors, and so forth [2], cell-based therapies are being sought for, as a promising approach to the condition. Several works of literature have reported the application of Bone Marrow Mononuclear Cell transplantation in Spinal Cord Injury with varying success rates [3–12]. It should be noted that CD34+ Hematopoietic Stem Cell (HSC) quantity is important because it has been reported that CD34 + cell quantity is an important dosage indicator for the success of BMMNC cell therapy [13]. Since clinical success of therapy might be attributed to the cell composition, an analysis of the same is needed to predict the success of such cell-based therapies. There are several works of literature on the composition of progenitor cell components in blood and bone marrow of healthy donors [14–16], but seldom in patients. In particular, the composition and other characteristics of bone marrow stem cells in spinal cord injury patients might be different from healthy individuals and even in patients with other kinds of organ dysfunctions, because spinal cord injury patients differ from the rest, in characteristics such as sedentary life style, body mass index, changes in neuronal control over hematopoiesis after the injury, and so forth [17–20]. This revelation can be drawn from works of literature like the study by Chernykh et al., which has compared the phenotypical and functional characteristics of bone marrow stem cells from spinal cord injury patients and healthy donors. In that study, it is stated that the percentage of CD34+CD38− hematopoietic stem cells is elevated in these patients compared to donors [21]. Also, Wright et al. published a study in which they examined the growth in cell culture of MSCs isolated from individuals with SCI, compared with non-SCI donors and they reported that age, level of spinal injury, and cell-seeding density were all related to the growth kinetics of MSC cultures *in vitro *[22].

In this study, we present a retrospective analysis of the data on BMMNC and HSC quantity obtained from 332 spinal cord injury patients admitted for autologous BMSC application over five years and we arrived at various indices such as BMMNC present in per mL of BM and percentage of CD34+ HSC in these patients.

## 2. Materials and Methods

### 2.1. Patients

All the procedures were carried in accordance with the local and national regulatory guidelines. The procedures followed were in accordance with the ethical standards described by the Helsinki Declaration.

Three hundred and thirty-two bone marrow samples were included in the study. The bone marrow samples were obtained from spinal cord injury patients who were admitted to various hospitals for autologous application of BMMNC, after ethics committee approvals from the respective hospitals and after proper informed consent. Males were predominant, with a total of 267 against 65 females. The age of the patients ranged from 1 to 76 years. The level of injury varied, ranging from C1 to S1 level. The samples were grouped into four based on the age: 0 to 20 years—Group I, 21–40 years—Group II, 41–60 years—Group III and 61–80 years—Group IV. The time from injury to stem cell application ranged from one month to twenty years. The samples were included only if the patients' vital parameters were in the normal physiological range and they did not have any other abnormalities in their blood forming system such as an associated autoimmune disease or malignancy.

### 2.2. Bone Marrow Aspiration and Cell Isolation

The cell processing was done in a single institute for all the five years. Ninety to hundred mL (average 95 mL) of bone marrow aspirated from the ileac crest in all the patients was transported in an anticoagulant solution under cold chain and on reaching the lab the samples were subjected to processing immediately. The samples were processed under cGMP SOP's class 10000 clean room and class 100 Biosafety cabinets. The samples were subjected to Ficoll gradient centrifugation procedure and the BMMNCs were collected by removing the buffy coat. The viability of the cells was checked using Trypan blue and cell count was done by using Neubaur's Haemocytometer. The quantity of BMMNC per mL was calculated. A portion of the isolated BMMNCs from each sample was sent for Immunophenotyping (IP Typing) analysis to analyze the quantity of CD34+ cells by flow cytometry (BD FACS Calibur, USA).

## 3. Results

**Figure 1:**
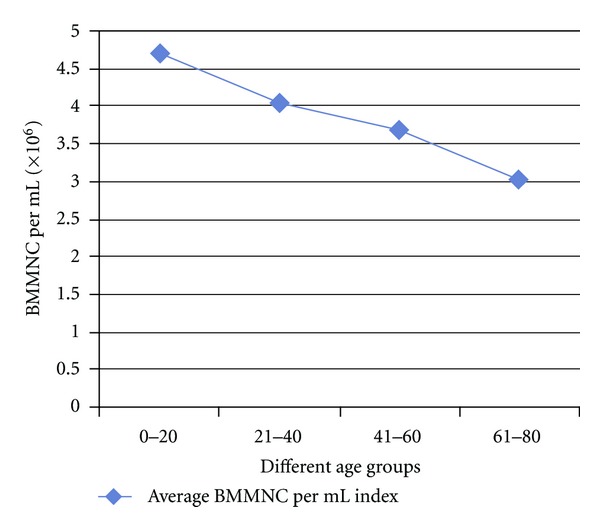
Average quantity of Bone Marrow Mononuclear Cells (BMMNC) per mL across various age groups of bone marrow samples from Spinal Cord Injury (SCI) victims.

**Figure 2:**
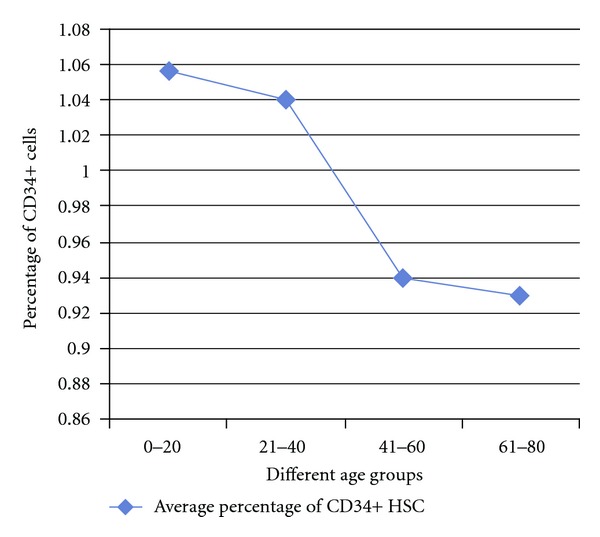
Average quantity of CD34+ Hematopoietic Stem Cell (HSC) percentage across various age groups of bone marrow samples from Spinal Cord Injury (SCI) victims.

The results are presented in Table 1. The average BMMNC per mL in patients of age group 0–20 years was 4.71 million; in 21–40 years it was 4.03 million; in 41–60 years it was 3.67 million; in 61–80 it was 3.02 million. The average BMMNC per mL in all the 332 patients ranged from a minimum of 1.47 million to a maximum of 15.36 million. The percentage of CD34+ cells in those patients belonging to the age group of 0–20 years was 1.05; in 21–40 year group 1.04; in 41–60 year group 0.94; in 61–80 year group 0.93. The average BMMNC per mL in males was 3.86 million, while in females it was 3.66 million. The average CD34% in males was 1.01, while in females it was 0.925. A slight decrease in the BMMNC per mL and CD34+ quantity was observed with increase in the age but they were not stastically significant (Figures 1 and 2). The decline in CD34+ was sharp between the groups 20–40 and 40–60, and particularly, females in the reproductive age group had a lesser CD34+ HSC and BMMNC quantity compared to males of similar age. Clinical observations of the patients till date showed that there are no adverse reactions in any of the patients and further followup is underway.

## 4. Discussion

Bone marrow mononuclear cell transplantation for spinal cord injury is a promising approach with several studies reporting varying efficacies in animal models and in humans. [3–12]. The mechanisms by which these cells contribute to spinal cord injury repair are still not understood to the fullest. The proposed mechanisms by which the injected cells may act are by transdifferentiation into neuronal lineage, inducing cells in the region neighbouring the spinal cord injury to regenerate or replace the injured neurons, secretion of neurotrophic factors, and altering the *in vivo* milieu in favour of regeneration [23]. Though BMMNC is a comprehensive cell population, particular importance has been attached to the CD34+ HSC quantity as several studies have reported that it is an important dosage indicator for success of bone marrow cell therapy [7–9].

There are several works of literature on the quantity of Mononuclear Cells (MNCs) and CD34+ HSCs in per mL or the whole bone marrow.  Table 2 gives the details of some of the literature on such parameters based on our search. It has been found that works of literature reporting such data are very limited, which can serve as a valuable reference on the quantity of BMMNC or CD34+ HSC across different age groups of individuals, especially in spinal cord injury patients. Chernykh et al. have reported a similar study in spinal cord injury patients but the sample size is limited [21]. Our study on samples obtained from 332 spinal cord injury victims can thus serve as a very valuable literature for future studies on evaluation of quantity of bone marrow for optimal cell isolation and dosing studies on stem cells in spinal cord injury.

Body mass index has a significant role to play in progenitor cell population and their mobilization with majority of the reports indicating that higher BMI and obesity are associated with increased CD34+ cell counts [16, 24, 25]. It is noticed that spinal cord injury victims generally have higher BMI due to their sedentary lifestyle and higher food intake [17–19]. Hence, it is logical to expect the CD34+ cell percentage to be higher in them. Another reason stated for higher CD34+ HSC count in spinal cord injury patients is the increase in the proliferative potential of CD34+ HSCs rising from the impaired innervation resulting in attenuation of negative control over HSC proliferation from the nervous system [21]. Spinal cord injury leads to secondary complications like alterations in lipid and glucose metabolism, which may lead to increased body fat. Chronic spinal cord injury also has been shown to increase the level of cytokines and interleukins thereby leading to increased inflammatory activity, which may also be a possible mechanism behind the increased CD34+ HSC proliferation in spinal cord injury. The increased progenitor levels in spinal cord injury may have a positive effect in improving tissue repair and regeneration in spinal cord injury following stem cell application [20]. A study in a chick embryo concluded that HSCs produce neurons more efficiently in a regenerating spinal cord due to favourable microenvironment [26]. All these studies imply that autologous HSCs from the spinal cord injury patients can be of a therapeutic advantage in these patients. However, increased inflammation combined with decreased immunity observed in spinal cord injury patients may also lead to increased risk of cancer incidence in these patients as reported [20]. The average BMMNC per mL and CD34+ HSC % obtained in the present study was 3.85 million, which is relatively less compared to that reported by Chernykh et al. [21], but the number of study subjects is substantially high in the present study. Also, the large difference in age, level of injury, and time of bone marrow harvest since time of injury between the patients may influence the average values obtained. The present study provides information of the index of quantity of BMMNC present per mL of bone marrow in different age groups of patients with spinal cord injury which is a worthy reference for future studies.

The influence of donor characteristics on the yield of BMMNC and the percentage of hematopoietic stem cells in the BMMNC population have been the objective of various studies described in works of literature [15, 16]. Variables such as gender, genetics, sleep, and circadian rhythm have been found to influence the quantity and other characteristics of BMMNC and CD34+ cells [27–31]. We have assessed the quantity of BMMNC and CD34+ HSCs in relation to age and gender in this study.

The slight decrease in CD34+ cell quantity with increasing age in our study, though statistically not significant (Figure 2), needs thorough analysis taking into consideration other parameters of significance, which are beyond the scope of this study. On the influence of age on BMMNC and CD34+ cell count, there are conflicting reports as in few of the works of literature; it has been reported that there is indeed a decrease in CD34+ cell quantity with increasing age [30, 31, 38–40], but few other works have indicated that though the functionality of HSC decreases with increasing age, there is not much difference in the HSC number with increasing age [41, 42] including reports that there is an increase in multipotent CD34(+) CD38(−) population in the bone marrow of elderly individuals above 70 years of age. Also, in the same study it was reported that CD34(+) CD38(+) CD90(−) CD45RA(+/−) CD10(−) and CD34(+) CD33(+) myeloid progenitors persist at the same level in the bone marrow, while the frequency of early CD34(+) CD38(+) CD90(−) CD45RA(+) CD10(+) and committed CD34(+) CD19(+) B-lymphoid progenitors decreases with age [43]. Cho et al. suggests that there are several subsets in HSCs, which are very different from each other, each possessing distinct self-renewal capacities, differentiation abilities, life span, and repopulation kinetics and with aging, lymphoid-biased HSCs are decreased, while the myeloid-biased HSCs accumulate, indicating that aging instead of affecting the HSC in general changes the clonal composition of the HSC compartment [44]. In another study, it was reported that in hematopoietic stem cell transplantation (HSCT), 0–20 year-old donors were yielding relatively higher Mesenchymal Stem Cells (MSCs) in shorter duration and their biological characteristics were superior to that of older age groups [40]. It should be understood that in our study the CD34+ cells as a whole have been studied and not the clonal proliferative capability. It has been reported that there is a strong genetic component that contributes to the changes in stem cell numbers during aging [30].  Thus, it will be ideal to analyse not only the CD34+ cell quantity as a whole, but also the clonal populations in the different age groups of such patients as it will serve as an accurate indicator of the variations in bone marrow functionality with increasing age. This analysis will help in predicting the success of cell transplantation in different age groups of patients with spinal cord injury.

**Figure 3:**
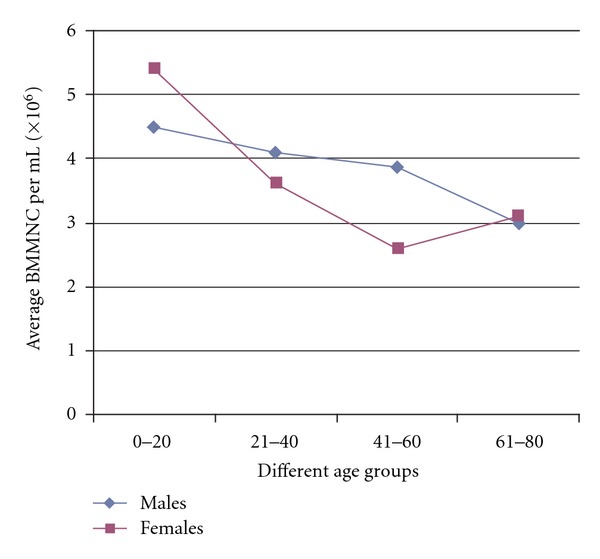
Comparison of average quantity of Bone Marrow Mononuclear Cells (BMMNC) per mL between male and female Spinal Cord Injury (SCI) victims.

**Figure 4:**
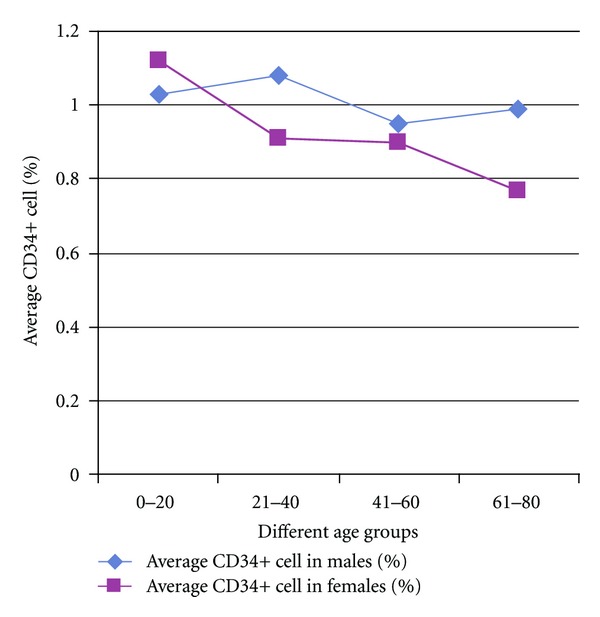
Comparison of CD34+ Hematopoietic Stem Cell (HSC) percentage between male and female Spinal Cord Injury (SCI) victims.

On the influence of gender on BMMNC and CD34+ HSC, in an article by Newman et al. on the yield of nucleated cells from marrow derived from cadaveric vertebral bodies, it has been reported that female donors yielded lower cell numbers independent of age and male donors less than 30 years of age yielded the highest number of cells [27]. There are also studies to show that male infants have significantly higher median CD34+ cell concentrations than female infants, which are reflected in an increased number of colony-forming cells, erythroblastic colonies, and granulocyte-macrophage colonies in their peripheral blood [45, 46]. It has also been suggested, based on literary evidence, that “17*β*-estradiol exerts negative influence on the production of B-lineage cells by modifying the differentiation, proliferation, and survival of early B-cell precursors and androgens exert an inhibitory effect on B lymphopoiesis but enhance erythropoietic differentiation and thrombocytopoiesis.” [46]. Thus, it can be understood that sex hormones may influence HSC and hematopoiesis but the effects of different sex hormones on individual cell populations of the bone marrow need further analysis. In our study, a steady decrease in the BMMNC per mL can be seen with increasing age but it is not of statistical significance. The decline in CD34+ was sharp between the 20–40 and the 40–60 age groups and particularly females in the reproductive age group had a lesser CD34 and BMMNC quantity compared to males though statistically insignificant. The lesser BMMNC per mL and CD34+ HSC in females compared to males might be due to the influence of sex hormones, which exert their effects on hematopoiesis in the bone marrow and this effect of female sex hormones will possibly be more pronounced in the reproductive age group of females appreciated by the sharp decline of BMMNC per mL and CD34+ HSC in Figures 3 and 4. However, the number of females is several times lesser than the number of males in each age group in this study and hence, further investigation on these lines is warranted in studies with equal number of samples from both the genders.

Clinical observations in the patients showed that there were no adverse reactions in any of the patients. The interim results of six-month followup on 108 patients out of these 332 patients revealed that “14.11% of patients reported at least 2 grades of improvement in motor power and 4.7% of patient were able to walk independently. 16.47% of patients reported subjective sensory improvement; none of the patients had abnormal sensations such as Allodynia and 9.41% of patients had improvement as documented by Urodynamic studies.” [47]. Since the main aim of the present study is evaluation of characteristics of the bone marrow in these patients, results of clinical evaluation will be out of scope of the current study.

The two indices described above, namely, the BMMNC index, that is, quantity of BMMNC per mL of bone marrow and the CD34+ cell index, that is, percentage of CD34+ cells in a given bone marrow sample can also be used for quantification studies to assess the approximate quantity of bone marrow to be harvested from spinal cord injury patients for therapeutic application. Though CD34+ cell quantity is widely used as a predictor of engraftment, a recent study done on 435 Cord Blood Transplants has suggested that the CFU dose is a better predictor of engraftment [48]. Further studies should be done on analysing this triad of parameters: the BMMNC per mL, CD34+ cell quantity per mL, and CFU in bone marrow samples in various age groups of patients with spinal cord injury also with healthy donors to arrive at data, based on which this triad can be made as a gold standard testing method in accurately predicting the functionality and quality of the bone marrow for application in spinal cord injuries.

## 5. Conclusion

We have described two useful indices for assessment of BMMNC and CD34+ HSC quantity in bone marrow based on data obtained from spinal cord injury patients with normal vital physiological parameters. In our evaluation, the average BMMNC per mL and the percentage of CD34+ cells show a decline with aging in spinal cord injury victims of both males and females. The BMMNC and CD34+ HSC are relatively lower in females than males and there is a sharp decline of CD34+ HSC in females in the reproductive age group. The fact that the characteristics of BMMNCs and HSCs will differ in spinal cord injury patients compared to normal patients due to the differences in lifestyle and other parameters makes these findings important, as the values of BMMNC and CD34+ HSC from this study may be used as a reference for future studies. The decreased BMMNC and CD34+ HSC in females will have to be analysed for their relevance to hormonal influence. The Colony Forming Unit (CFU) analysis, which is more relevant as physiological indicator, when assessed may throw further light and add significance.

## Figures and Tables

**Table 1:** Age and genderwise quantity of Bone Marrow Mononuclear Cells (BMMNC) and percentage of CD34+ cells in Spinal Cord Injury (SCI) victims.

Groups	Age (years)	No. of patients	No. of males	No. of females	Average quantity of BM-aspirated (mL)	Average quantity of BMMNC isolated (×10^6^)	Average CD34+ HSC%	Average BMMNC per mL index	Average CD34+ HSC% in males	Average CD34+ HSC% in females	Average BMMNC per mL index in males	Average BMMNC per mL index in females
Group I	0–20	35	24	11	92	434	1.05	4.71	1.02	1.12	4.5	5.39
Group II	21–40	203	165	38	100	403	1.04	4.03	1.08	0.91	4.1	3.6
Group III	41–60	83	70	13	100	367	0.94	3.67	0.95	0.9	3.87	2.59
Group IV	61–80	11	8	3	100	302	0.93	3.02	0.99	0.77	3	3.09

**Table 2:** Data of the quantity of Bone Marrow Mononuclear Cells (BMMNC) and CD34+ Hematopoietic Stem Cells (HSC) from the literatures based on our search.

S. No.	Author	Year of Publication	No. of Samples	Patients or Donors	Parameters assessed	Mean Quantity of MNC/ml	Mean CD34%	Mean CD34+ cell count
1	Ema et al. [32]	1990	12	Donors	CD34%	NA	1.05% ± 0.44%^∗^	
2	Chernykh et al. [21]	2006	10	Donors	MNC, count and CD34%	7.5 ± 2.2 × 10^6^	5.40 ± 1.35	
3	Chernykh et al. [21]	2006	16	Spinal Cord injury Patients	MNC count and CD34 percentage	11.0 ± 1.1 × 10^6^	5.4 ± 0.6	
4	Mohamadnejad et al. [33]	2007	4	Liver Cirrhosis patients	MNC and CD34 percentage	3.13 × 10^8^		5.25 × 10^6^
5	Hernández et al. [34]	2007	12	Critical limb Ischemia patients	MNC and CD34+ cell counts	1.74 ± 1.23 × 10^9^ in group A (Separation done using blood cell separator) and 2.47 ± 1.48 × 10^9^ in group B (Separation done by density gradient by Ficoll-Hypaque)		8.14 ± 6.67 × 10^7^ in group A and 7.90 ± 5.46 × 10^7^ in group B
6	Kaparthi et al. [35]	2008	5	Cardiac Disease patients	MNC and CD34+ cell count and percentage	9.16 × 10^7^	0.348	3.68 × 10^5^
7	Harting et al. [36]	2009	36	10 paediatric and 26 adult Non-cancer patients	MNC counts	Paediatric patients −2.1 × 10^6^/mL and in older patients −3.2 × 10^6^/mL		
8	Zhang et al. [16]	2010	104	Donors	CD34+ cell count and Circulating Immature Cells (CIC) count	CIC = 9*·*4 (4*·*3–21*·*1) × 10^9^ L^−1^		Total CD34+ cell count (×10^6^) is 395*·*7 (102–1282)
9	Perseghin and Incontri [37]	2010	10	Patients-nine with chronic GvHD and one with bullous pemphigoid	MNC	5.9 ± 2.19 × 10^9^ in the separation done by Spectra cell separator and 5.29 ± 2.39 × 10^9^ in the separation done by Amicus cell separator		

^∗^% mentioned is that of gated cells.
